# Changes in the community composition and function of the rhizosphere microbiome in tobacco plants with *Fusarium* root rot

**DOI:** 10.3389/fmicb.2025.1512694

**Published:** 2025-04-09

**Authors:** Min Yang, Yongzhan Cai, Tao Bai, Xiaonv Han, Rong Zeng, Dongmei Liu, Tao Liu, Rui Liu, Chan Ma, Lei Yu

**Affiliations:** ^1^College of Agronomy, Yunnan Urban Agricultural Engineering and Technological Research Center, Kunming University, Kunming, Yunnan, China; ^2^Qujing Branch of Yunnan Provincial Tobacco Company, Qujing, Yunnan, China; ^3^Zhanyi Agricultural Technique Extension Center, Qujing, Yunnan, China

**Keywords:** soil-borne fungal disease, *Fusarium* spp., microbial diversity, microbial interactions, rhizosphere

## Abstract

**Introduction:**

Tobacco root rot caused by *Fusarium* spp. is a soil-borne vascular disease that severely affects tobacco production worldwide. To date, the community composition and functional shifts of the rhizosphere microbiome in tobacco plants infected with *Fusarium* root rot remain poorly understood.

**Methods:**

In this study, we analyzed the differences in the compositions and functions of the bacterial and fungal communities in the rhizosphere and root endosphere of healthy tobacco plants and tobacco with *Fusarium* root rot using amplicon sequencing and metagenomic sequencing.

**Results and discussion:**

Our results showed that *Fusarium* root rot disrupted the stability of bacteria–fungi interkingdom networks and reduced the network complexity. Compared to healthy tobacco plants, the Chao1 index of bacterial communities in the rhizosphere soil of diseased plants increased by 4.09% (*P* < 0.05), while the Shannon and Chao1 indices of fungal communities decreased by 13.87 and 8.17%, respectively (*P* < 0.05). In the root tissues of diseased plants, the Shannon index of bacterial and fungal communities decreased by 17.71–27.05% (*P* < 0.05). Additionally, we observed that the rhizosphere microbial community of diseased tobacco plants shifted toward a pathological combination, with a significant increase in the relative abundance of harmful microbes such as *Alternaria, Fusarium*, and *Filobasidium* (89.46–921.29%) and a notable decrease in the relative abundance of beneficial microbes such as *Lysobacter, Streptomyces, Mortierella*, and *Penicillium* (48.48–81.56%). Metagenomic analysis further revealed that the tobacco rhizosphere microbial communities of diseased plants played a significant role in basic biological metabolism, energy production and conversion, signal transduction, and N metabolism, but their functions involved in C metabolism were significantly weakened. Our findings provide new insights into the changes in and interactions within the rhizosphere and root endosphere microbiomes of tobacco plants under the stress of *Fusarium* soil-borne fungal pathogens, while laying the foundation for the exploration, development, and utilization of beneficial microbial resources in healthy tobacco plants in the future.

## 1 Introduction

Tobacco root rot caused by *Fusarium* spp. is a global soil-borne vascular disease and occurs frequently in China's main tobacco-producing areas such as Yunnan, Guizhou, Sichuan, and Hunan (Berruezo et al., 2018; Yang et al., 2020; Tan et al., 2022). Pathogens usually invade the roots of tobacco plants and damage vascular bundles, leading to blackened necrotic vascular bundles and root rot, severely impairing the uptake of essential nutrients by plants (Gao et al., 2021). After *Fusarium* infection, the leaves of diseased tobacco plants turn yellow, the stems blacken, and in severe cases, the whole plant withers and dies, significantly reducing both the yield and quality of tobacco leaves (Lievens et al., 2008; Berruezo et al., 2018). Unfortunately, our preliminary survey revealed that Qujing, the largest tobacco-growing area in Yunnan Province, is hardest hit by *Fusarium* root rot, with an incidence rate as high as 15.25–32.10% and the main pathogen being *F. solani* (Gai et al., 2023; Zhao et al., 2023).

*Fusarium* is a globally distributed fungal genus, many species of which are plant pathogens that cause a wide range of damage. Two of these species, *F. graminearum* and *F. oxysporum*, rank fourth and fifth among the top 10 plant pathogenic fungi (Dean et al., 2012). Importantly, *Fusarium* species often overwinter as mycelia or chlamydospores in soil and cultivation medium or attached to seeds and can survive as saprophytes. Consequently, they can persist in soil for long periods and infect plants by colonizing xylem tissues through the roots under favorable conditions, making disease management nearly impossible. Pathogen attacks are constant, yet certain soils can suppress plant diseases even in the presence of pathogens. This ability is at least partly provided by the plant's root-associated microbial communities (Weller et al., 2002). For example, the symbiotic bacterial communities in *Arabidopsis thaliana* roots can shape the community structure of fungi and oomycetes, protecting plants from their attack (Durán et al., 2018). Therefore, the regulation of natural microbial communities is considered one of the most promising strategies for improving soil health to achieve comprehensive and sustainable disease management.

Plant roots are inhabited by many microbial communities, mainly categorized as rhizosphere and root endosphere microbial communities according to their habitat location. They colonize around and within plant roots, respectively, and play a critical role in resisting soil-borne pathogens, improving plant nutrient absorption, enhancing plant stress resistance, and regulating host immunity. When plants are attacked by pathogens, they can send a “cry for help” to the root microbiome, thereby selectively enriching plant-protective microbes (Yin et al., 2021). This protective effect also extends to subsequent generations of plants, known as the soil-borne legacy (Raaijmakers and Mazzola, 2016; Bakker et al., 2018; Zhuang et al., 2024; Ma et al., 2025). In view of the above, plant resistance to pathogens is achieved at least in part by recruiting beneficial microbes from the soil. In recent years, many studies have analyzed the dynamics of plant rhizosphere microbial communities under infection by soil-borne bacterial pathogens, demonstrating that related microbial communities help plants resist pathogen invasion through mechanisms such as niche competition, antibiotic secretion, and induced systemic resistance (Beneduzi et al., 2012; Schulz-Bohm et al., 2018; Yuan et al., 2018; Trivedi et al., 2020; Ahmed et al., 2022). However, the role of root endosphere microbial communities in resisting infection by soil-borne pathogens is often overlooked, and few studies have combined rhizosphere and root endosphere microbial communities to reveal their interactions with plants and soil-borne pathogens, especially against soil-borne fungal pathogens such as *Fusarium* (Kwak et al., 2018; Carrión et al., 2019; Zhang et al., 2021).

With the development of high-throughput sequencing technology, culture-independent amplicon sequencing has enabled unprecedented breadth and precision in understanding the composition of microbial communities; in addition, the development of metagenomic technology has allowed us to understand the function of microbial communities from a systems perspective (Berg et al., 2020; Liu et al., 2021). In various ecological environments, interactions between microbial species can form large and complex functional networks through the exchange of energy, metabolites, and signals (Deng et al., 2012; Peng et al., 2024). It is critical to reveal and understand the structure, dynamics, and potential mechanisms of ecological networks for the study of plant microecosystems (Coyte et al., 2015). Therefore, the combination of amplicon sequencing, metagenomic sequencing, and microbial co-occurrence network analysis facilitates a more comprehensive understanding of microbial communities dynamics while further revealing the functional potential of microbial taxa, including their potential interactions with the environment and other organisms. Previous studies have revealed that the rhizosphere soil and endophytic fungal communities of tobacco plants infected by the pathogenic fungus *F. oxysporum* exhibit more complex network structures and stronger interaction relationships compared to those of healthy plants. However, this study focused solely on the changes in the rhizosphere and root endosphere fungal communities (Tan et al., 2022).

In this study, we hypothesize that *F. solani* infection will lead to simultaneous changes in the bacterial and fungal communities within the rhizosphere soil and root tissues of tobacco plants, thereby increasing the differences in the composition of the rhizosphere microbiome between diseased and healthy tobacco plants. Additionally, considering that the ecological functions of plants, including pathogen defense, are achieved through the synergistic interactions of the plant microbiome, we also hypothesize that the functions of the associated microbial communities in the rhizosphere and root endosphere of tobacco plants may differ between diseased and healthy states. To test these hypotheses, we collected rhizosphere soil and root tissue samples from tobacco plants with *Fusarium* root rot disease and healthy plants in the same plot. Using amplicon (bacterial and fungal) and metagenomic sequencing technologies, we explored the compositional and functional differences in the rhizosphere and root endosphere microbial communities in the diseased and healthy tobacco plants, followed by further analysis of the interactions of related microbial communities through microbial co-occurrence network analysis. Our study results help elucidate the changes in the rhizosphere and root endosphere microbial communities in tobacco plants under the stress of soil-borne fungal pathogens, reveal the interactions between rhizosphere and root endosphere microbial communities and pathogenic fungi, and are of great significance for utilizing rhizosphere microbial communities to enhance plant health and maximize crop yield.

## 2 Materials and methods

### 2.1 Sample collection

**Figure 1:**
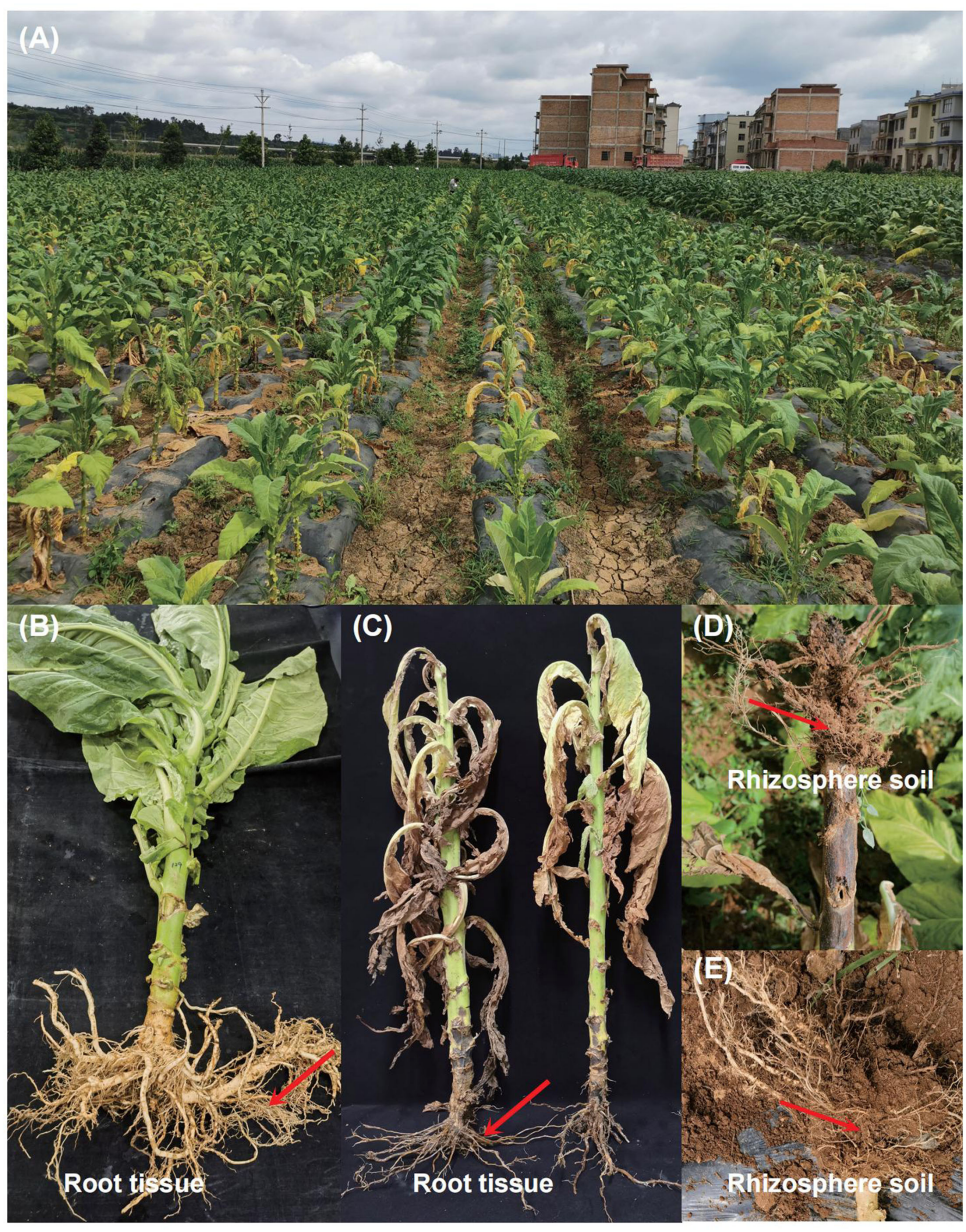
Field symptoms of tobacco *Fusarium* root rot and sample collection. **(A)** After being infected with *Fusarium*, the leaves of diseased tobacco plants turn yellow, the stems turn black, and in severe cases, the whole plant wilts and dies. **(B)** Healthy flue-cured tobacco plants with green leaves and white-colored roots. **(C)** Disease flue-cured tobacco plants with root rot symptoms (such as yellowing, and wilting of leaves, and necrotic lesions on the stem and root). Root tissues from healthy and diseased tobacco plants were cut off respectively to be used as root tissue samples. **(D, E)** After removing the bulk soil, a brush was used to carefully collect the tiny soil particles attached to the roots of healthy and diseased tobacco plants as rhizosphere soil. The red arrow points to our sampling location.

The test samples were collected in July 2023 from the Yunyan 100 flue-cured tobacco planting area in Dali Shu Village, Yuezhou Town, Qilin County, Qujing City, Yunnan Province (103°52′05″E, 25°14′ 55″N). In the same plot (with similar natural conditions, soil properties, and consistent field management practices), six tobacco plants with typical symptoms of *Fusarium* root rot and six healthy tobacco plants were selected for rhizosphere soil and root sample collection (Figure 1). To collect the samples, upper 2–3 cm layer of soil was first removed, and tobacco plants were uprooted. The adhered bulk soil was shaken off, and the soil tightly adhering to the root surface was collected with a brush as the rhizosphere soil samples. The rhizosphere soil samples were named S_D (rhizosphere soil of diseased plants) and S_H (rhizosphere soil of healthy plants). After the rhizosphere soil was collected, the fibrous roots were cut and classified as root tissue samples, which were named R_D (root tissue of diseased plants) and R_H (root tissue of healthy plants). The experiment consisted of four treatments, each with six replicates (each replicate derived from an individual tobacco plant), totaling 24 samples, including 12 rhizosphere soil samples and 12 root tissue samples.

The soil samples were analyzed for various physicochemical properties using standardized methods: after being suspended in water (soil:water = 1:2.5, w/v), the soil pH was measured using a pH meter (Wang et al., 2017). Soil organic matter, as well as available N, P, and K, were measured using the methods described by Cai et al. (2021) (Supplementary Table 1). In order to avoid contamination by environmental microorganisms, we carried out surface sterilization on the collected root tissue samples. The specific procedures were as follows: rinsed with a large amount of sterile distilled water for 5 min to remove the soil attached to the root tissue, soaked in 75% ethanol for 30 s, rinsed with sterile distilled water three times (5 min each time), soaked in 3% sodium hypochlorite solution for 3 min, and then rinsed with sterile distilled water three times (5 min each time). The surface-sterilized root tissues were cut into small pieces with an aseptic surgical knife in the ultra-clean workbench, were quickly frozen in liquid nitrogen, and were immediately stored in a −80°C refrigerator for later use.

### 2.2 Soil microbial DNA extraction and amplicon sequencing

Total genomic DNA was extracted from 12 rhizosphere soil samples following the procedures of the DNeasy^®^ PowerSoil^®^ Pro Kit (Qiagen, Hilden, Germany). The concentration and purity of the extracted DNA were measured using NanoDrop 2000, and the quality of DNA extraction was evaluated by 1% agarose gel electrophoresis. With the DNA extracted as described above as a template, two pairs of universal primers, 338F (5′-ACTCCTACGGGAGGCAGCAG-3′) and 806R (5′-GGACTACHVGGGTWTCTAAT-3′), as well as ITS1 F (5″-CTTGGTCATTTAGAGGAAGTAA-3′) and ITS2 R (5′-GCTGCGTTCTTCATCGATGC-3′) (Zhang et al., 2024), were employed to amplify the V3-V4 variable region of the bacterial 16S rRNA gene and the fungal ITS sequence, respectively. Amplicon libraries were sequenced on the Illumina Miseq PE300 platform in Shanghai Majorbio Bio-Pharm Technology Co., Ltd.

### 2.3 Root tissues endophytic microbial DNA extraction and amplicon sequencing

In accordance with the kit instructions, the DNA of 12 root tissue samples was extracted respectively by following the procedures of the DNeasy^®^ PowerSoil^®^ Pro Kit (Qiagen, Hilden, Germany). The concentration and purity of the DNA were measured using NanoDrop 2000, and the quality of DNA extraction was examined by 1% agarose gel electrophoresis. The endophytic fungal diversity was analyzed using the universal primers ITS1 F (5′-CTTGGTCATTTAGAGGAAGTAA-3′) and ITS2 R (5′-GCTGCGTTCTTCATCGATGC-3′) (Adams et al., 2013; Yang et al., 2024). In the analysis of bacterial communities, a two-step PCR amplification was performed, with bacterial primers 799F (5′-AACMGGATTAGATACCCKG-3′)/1392R (5′-ACGGGCGGTGTGTRC-3′) and 799F (5′-AACMGGATTAGATACCCKG-3′)/1193R (5′-ACGTCATCCCCACCTTCC-3′) used for 16S rRNA gene amplification, respectively (Wang et al., 2021; Yang et al., 2024). The PCR products were recovered using 2% agarose gel and further purified using the AxyPrep DNA Gel Extraction Kit (Axygen Biosciences, Union City, CA, USA) according to the manufacturer's instructions. Amplicon libraries were sequenced on the Illumina Miseq PE300 platform in Shanghai Majorbio Bio-Pharm Technology Co., Ltd.

### 2.4 Analysis of amplicon sequencing data

The raw sequencing reads were quality-controlled using Fastp (v 0.19.6) (Chen et al., 2018), and then the paired reads were merged into a single sequence using FLASH software (v 1.2.11) (Magoč and Salzberg, 2011). The processed high-quality sequencing reads were clustered into operational taxonomic units (OTUs) by UPARSE software (v 11) at a 97% similarity threshold (Edgar, 2013). For taxonomic information, species annotation for OTUs was performed using the RDP classifier (v 2.13) in the SILVA 16S rRNA gene database (v 138) (Quast et al., 2013) and the UNITE Fungal ITS database (v 8.0) (Kõljalg et al., 2005) with a confidence threshold of 70%. The relative abundances of species were analyzed at the phylum and genus levels. Alpha diversity indices, including the Shannon and Chao 1 indices, were calculated by Mothur software (v 1.30.2). Principal coordinate analysis (PCoA) based on the Bray–Curtis distance algorithm was carried out to examine the overall changes in microbial community structure among the samples, and the permutational multivariate analysis of variance (PERMANOVA) statistical tests were employed to analyze whether the differences in microbial community structure between sample groups were significant (Oksanen et al., 2007). Co-occurrence network analysis of bacterial and fungal communities was performed using the SparCC method (correlation coefficient > 0.75, *P* < 0.05) on the integrated network analysis pipeline (iNAP, https://github.com/yedeng-lab/iNAP) (Feng et al., 2022). The networks were visualized using the interactive platform Gephi (Bastian et al., 2009).

### 2.5 Soil microbial metagenomic sequencing

To further characterize the relevant functions of the rhizosphere soil microbial communities of tobacco plants in diseased and healthy states, we selected three DNA samples each from the rhizosphere soil of diseased and healthy tobacco plants extracted in section 1.2 for metagenomic sequencing. First, the DNA concentration and purity were measured, and the integrity of the DNA was detected by 1% agarose gel electrophoresis. Then, DNA extract was fragmented to an average size of about 400 bp using Covaris M220 (Gene Company Limited, China) for paired-end library construction. A paired-end library was constructed using NEXTFLEX Rapid DNA-Seq (Bioo Scientific, Austin, TX, USA). Adapters containing the full complement of sequencing primer hybridization sites were ligated to the blunt-end of fragments. Paired-end sequencing was performed on Illumina Novaseq 6000 (Illumina Inc., San Diego, CA, USA) at Shanghai Majorbio Bio-Pharm Technology Co., Ltd.

### 2.6 Analysis of metagenomic sequencing data

Raw sequences were quality-filtered using fastp, and reads with a length < 50 bp, an average base quality below 20, or the presence of N bases were removed. The filtered sequences were assembled using MEGAHIT (v 1.1.2) (Li et al., 2015), and contigs with a length ≥ 300 bp were selected as the final assembly results. Open reading frames (ORFs) were predicted for the assembled contigs using Prodigal (v 2.6.3) (Hyatt et al., 2010). Genes with nucleic acid lengths greater than or equal to 100 bp were selected, and the gene sequences predicted from all samples were clustered using CD-HIT software (v 4.6.1) (Fu et al., 2012). The longest gene in each class was taken as the representative sequence to construct a non-redundant gene set. The amino acid sequences of the non-redundant gene set were compared with those in the NR database (v nr_202209) using Diamond (v 2.0.13), and the species annotations were obtained from the taxonomic information database corresponding to the NR database. Reads after quality control were mapped to the non- redundant gene catalog with 95% identity using SOAPaligner (v 2.21) (Li et al., 2008), and gene abundances in each sample were evaluated. Read counts were normalized to per kilobase per million mapped reads (RPKM) using CoverM (v 0.4.0). Functional profiles including KEGG Orthology (KO), Cluster of orthologous groups of proteins (COG) and Carbohydrate-active enzymes (CAZyome) annotations were conducted using Diamond against the Kyoto Encyclopedia of Genes and Genomes database (v 202109), COG database (v 2020) and CAZy database (v 8).

### 2.7 Statistical analysis

The non-parametric Wilcoxon rank-sum test was used to assess differences in the average relative abundances of the same bacterial and fungal species between the diseased and healthy groups. Significant differences in Alpha diversity indices (e.g., Shannon index and Chao1 index) among treatments were determined using Duncan's multiple range test at *P* < 0.05 in IBM SPSS version 20.0. All figures were adjusted, combined, and modified using Adobe Illustrator 2020.

## 3 Results

### 3.1 Microbiome resources in the rhizosphere of tobacco in diseased and healthy states

To understand the differences in the rhizosphere soil and root endophytic microbial communities of tobacco plants in diseased and healthy states, 532,546 valid bacterial sequences and 559,425 valid fungal sequences were obtained from 12 tobacco rhizosphere soil samples through the amplification of bacterial/fungal 16S rRNA (V3-V4)/ITS, respectively, with an average length of 416 bps and 237 bps respectively (Supplementary Table 2). 474,562 valid bacterial sequences and 446,381 valid fungal sequences were obtained from 12 tobacco root samples, with an average length of 376 bps and 234 bps respectively (Supplementary Table 3). The rarefaction curves generated from OTUs indicate that the rarefaction of bacterial and fungal communities in different treatment groups has reached a reasonable number of reads in all samples (Supplementary Figure 1).

### 3.2 Rhizosphere microbiome diversity of diseased and healthy tobacco

**Figure 2:**
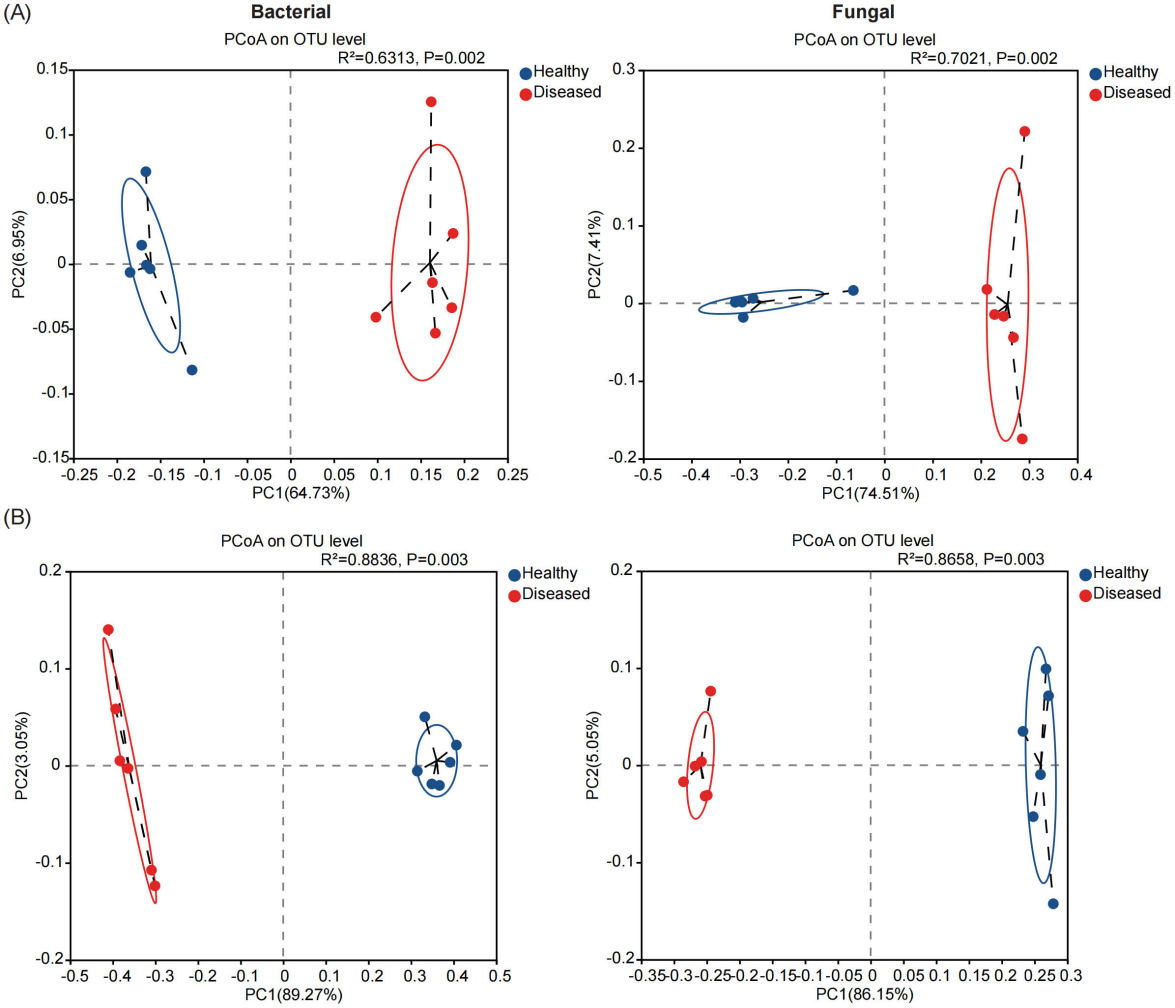
The microbial community structures (Beta - diversity) in the rhizosphere soil **(A)** and root endosphere **(B)** of tobacco plants under diseased and healthy conditions. Principal coordinate analysis (PCoA) plots based on the Bray-Curtis dissimilarity matrices with permutational analysis of variance (PERMANOVA), showing the changes in the structure of the bacterial (left) and fungal (right) community composition.

We evaluated the microbial community structure (beta diversity) in the rhizosphere soil and root endosphere of diseased and healthy tobacco plants using principal coordinate analysis (PCoA) based on the Bray–Curtis distance algorithm. Permutational analysis of variance (PERMANOVA) showed that *Fusarium* root rot had a greater effect on endophytic bacterial and fungal communities in the tobacco roots than in the rhizosphere soil (roots: *R*^2^ = 0.8836 for bacteria and *R*^2^ = 0.8658 for fungi; rhizosphere soil: *R*^2^ = 0.6313 for bacteria and *R*^2^ = 0.7021 for fungi; *P* < 0.01 for both). We further found that the rhizosphere and endophytic bacterial and fungal communities in the roots of diseased and healthy tobacco plants were separated on the first principal component. The rhizosphere soil samples and root samples from healthy and diseased tobacco plants were distributed on the left and right sides of the PC1 axis, respectively (Figures 2A, B). Overall, the rhizosphere and endophytic microbial communities of the healthy and diseased tobacco plants formed different clusters, suggesting that the disease state is likely the largest source of variation in the tobacco rhizosphere microbiome.

We further evaluated the changes in the alpha diversity indices (Shannon and Chao 1) of the rhizosphere microbiome of the diseased and healthy tobacco plants. First, we observed that the diversity and abundance indices of the bacterial and fungal communities in the tobacco rhizosphere soil were significantly higher than those in the root tissue samples. For the bacterial communities, the Shannon and Chao 1 indices of the rhizosphere soil samples from the diseased plants (S_D) were higher than those from the healthy plants (S_H). The Chao 1 index of the S_D samples was 4.09% higher (*P* < 0.05) than that of the S_H samples. In contrast, the Shannon and Chao 1 indices of the bacterial communities in the root tissue samples from the diseased plants (R_D) were lower than those in the root tissue samples from the healthy plants (R_H); in particular, the Shannon index in R_D was 27.05% lower than that in R_H (P < 0.05) (Supplementary Figure 2A). The diversity and abundance indices of the fungal communities in the rhizosphere soil of diseased tobacco plants were significantly lower than those in the rhizosphere soil samples from the healthy plants. The Shannon and Chao 1 indices of the S_D samples were 13.87 and 8.17% lower (*P* < 0.05), respectively, than those of the S_H samples. Moreover, the Shannon index of the fungal communities in the diseased root tissues was significantly reduced by 17.71% compared to the healthy root tissue samples, but the difference in the Chao 1 index was not significant (Supplementary Figure 2B).

### 3.3 Composition of the microbial community in the rhizosphere soil of diseased and healthy tobacco plants

**Figure 3:**
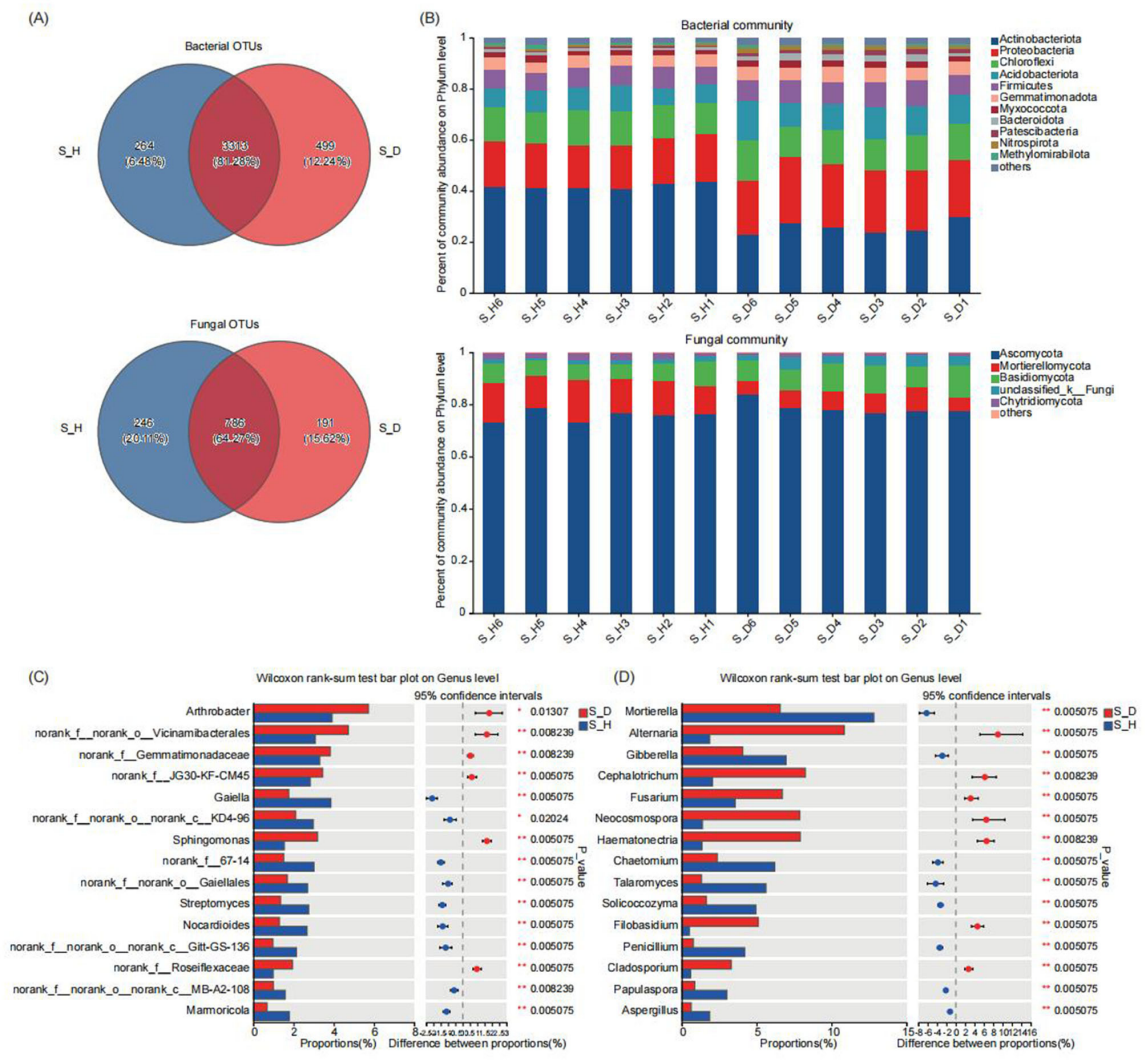
Microbial community composition in tobacco plants rhizosphere soil under diseased and healthy conditions. **(A)** The number of unique, shared, and common bacterial and fungal operational taxonomic units at different groups. **(B)** The bar plots of relative abundance illustrate the composition of bacterial (up) and fungal (down) communities in the rhizosphere soil at the phylum level under diseased and healthy conditions. Low abundance genera with less than 1% of the total sequences across all samples are grouped into “Other”. **(C, D)** The non-parametric Wilcoxon rank-sum test shows the differences in the average relative abundances of the same bacterial **(C)** and fungal **(D)** species (at the genus level) between the diseased group and the healthy group. * Stands for 0.01 ≤ *p* < 0.05, and ** stands for 0.001 ≤ *p* < 0.01.

Classification annotation was performed on the valid reads at the 97% clustering level. A total of 4076 bacterial OTUs and 1223 fungal OTUs were annotated from the 12 rhizosphere soil samples. The Venn diagram in Figure 3A shows the number of shared and unique OTUs across the different sample groups. There were 3,313 bacterial OTUs and 786 fungal OTUs shared among the different groups. The number of unique bacterial OTUs in the rhizosphere soil of diseased tobacco plants was higher than that in the healthy plants, but the number of unique fungal OTUs in the diseased plants was lower than that in the healthy plants. Next, we analyzed the species composition of the bacterial and fungal communities in the tobacco rhizosphere soil at different taxonomic levels. At the phylum level, Actinobacteria, Proteobacteria, Chloroflexi, Acidobacteriota, and Firmicutes were the dominant bacterial communities, and Ascomycota, Mortierellomycota, and Basidiomycota, were the dominant fungal communities in the tobacco rhizosphere soil (Figure 3B). Compared with those in the rhizosphere soil of the healthy tobacco plants, the relative abundances of Proteobacteria, Acidobacteriota, Firmicutes, and Gemmatimonadota in the rhizosphere soil of the diseased tobacco plants were all higher, whereas the relative abundance of Actinobacteria was significantly lower (*P* < 0.05) (Supplementary Table 4). The relative abundances of Ascomycota and Basidiomycota in the fungal communities were also significantly higher in the rhizosphere soil of the diseased tobacco plants than in that of the healthy plants, whereas the relative abundances of *Mortierellomycota* and *Chytridiomycota* were significantly lower (Supplementary Table 5).

At the genus level, *Arthrobacter, Bacillus, Gaiella, Sphingomonas, Streptomyces*, and *Nocardioides* were the most important bacterial communities, and *Mortierella, Alternaria, Gibberella, Cephalotrichum*, and *Fusarium* were the most important fungal communities in the tobacco rhizosphere soil (Supplementary Figure 3). The significance of the differences in bacterial and fungal taxa between the rhizosphere soils of the diseased and healthy tobacco plants was determined by the non-parametric Wilcoxon rank sum test. The multispecies difference test histograms are shown in Figures 3C, D. Among the top 15 species in relative abundance, compared with those in the rhizosphere soil of healthy plants, the relative abundances of *Arthrobacter* and *Sphingomonas* in the rhizosphere soil of diseased tobacco plants were significantly higher, whereas the relative abundances of *Gaiella, Streptomyces*, and *Nocardioides* were significantly lower (*P* < 0.05) (Figure 3C). Similarly, the relative abundances of fungal species such as *Alternaria, Cephalotrichum, Fusarium, Neocosmospora, Hematonectria, Filobasidium*, and *Cladosporium* were significantly higher in the rhizosphere soil of the diseased tobacco plants than in that of the healthy plants, whereas the relative abundances of *Mortierella, Gibberella, Chaetomium, Talaromyces, Solicoccozyma, Penicillium, Papulaspora*, and *Aspergillus* were significantly lower (*P* < 0.05) (Figure 3D). In particular, the relative abundances of *Alternaria, Fusarium, Filobasidium*, and *Cladosporium* in the S_D group were 483.92, 89.46, 921.29, and 452.64% higher than those in the S_H group, respectively, whereas the relative abundances of beneficial fungi such as *Mortierella, Talaromyces*, and *Penicillium* in the S_D group were 48.48, 76.71, and 81.56% lower than those in the S_H group, respectively. These results indicate significant differences in the dominant species and the relative abundances of the bacterial and fungal communities in the rhizosphere soil of diseased and healthy tobacco plants.

### 3.4 Composition of the endophytic microbial community in the roots of diseased and healthy tobacco plants

**Figure 4:**
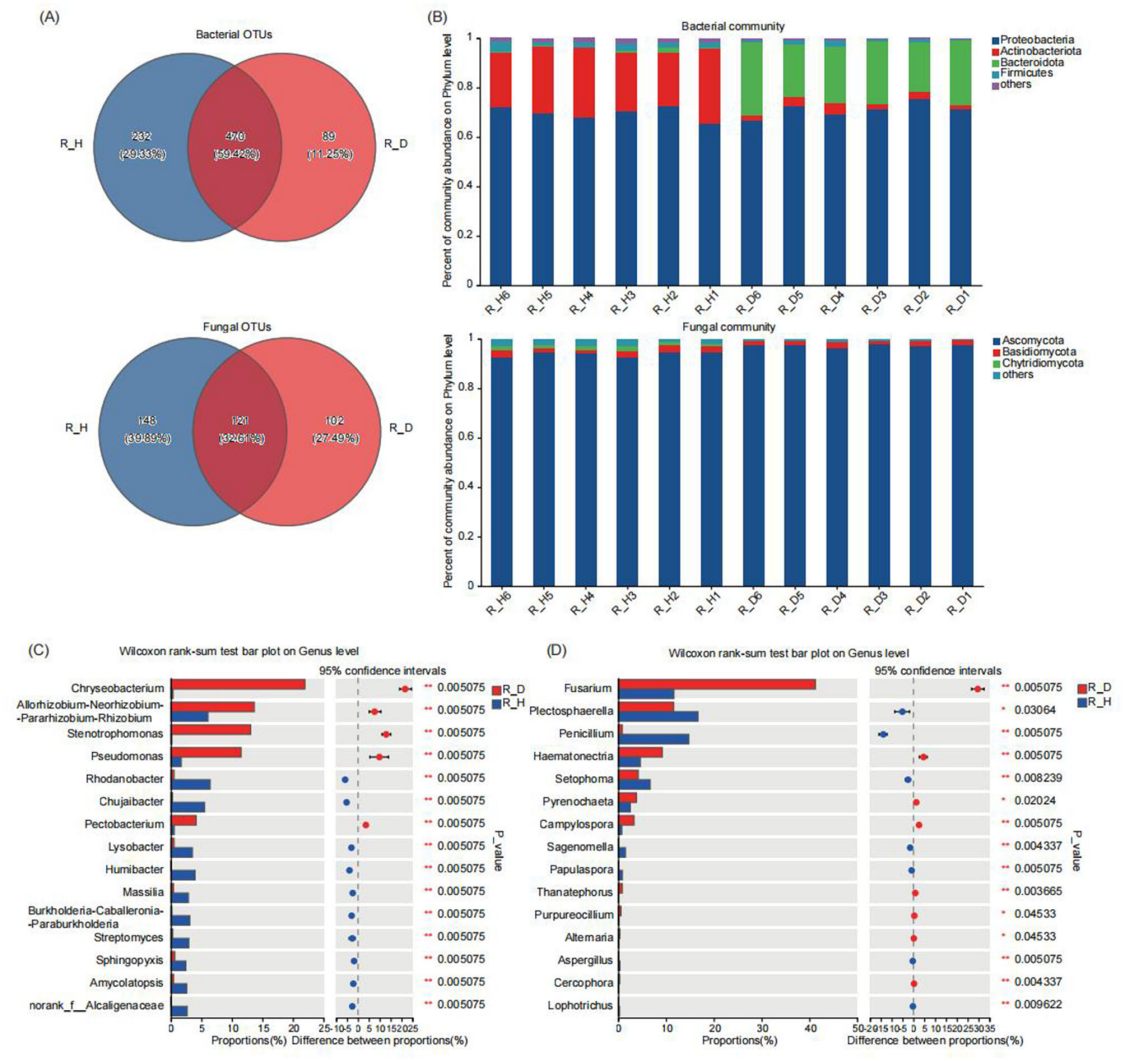
Microbial community composition in tobacco plants root endosphere under diseased and healthy conditions. **(A)** The number of unique, shared, and common bacterial and fungal operational taxonomic units at different groups. **(B)** The bar plots of relative abundance illustrate the composition of bacterial (up) and fungal (down) communities in the root endosphere of tobacco plants at the phylum level under diseased and healthy conditions. Low abundance genera with less than 1% of the total sequences across all samples are grouped into “Other”. **(C, D)** The non-parametric Wilcoxon rank-sum test shows the differences in the average relative abundances of the same bacterial **(C)** and fungal **(D)** species (at the genus level) between the diseased group and the healthy group. * Stands for 0.01 ≤ *p* < 0.05, and ** stands for 0.001 ≤ *p* < 0.01.

At the 97% clustering level, a total of 791 bacterial OTUs and 371 fungal OTUs were annotated from the 12 tobacco root samples, with 470 bacterial and 121 fungal OTUs shared among the different sample groups (Figure 4A). The number of unique bacterial and fungal OTUs in the root tissues of the diseased tobacco plants was significantly lower than in those of the healthy tobacco plants. Further analysis of the composition of the bacterial and fungal communities in the root tissues of the diseased and healthy tobacco plants at different taxonomic levels showed that at the phylum level, Proteobacteria, Actinobacteria, Bacteroidota, and Firmicutes were the main dominant bacterial phyla, and Ascomycota and Basidiomycota were the main dominant fungal phyla (Figure 4B). The relative abundances of Actinobacteria and Firmicutes in the root tissues of the diseased plants were significantly lower than those in the root tissues of the healthy plants; the relative abundances of these bacteria in the R_D group were 88.96 and 49.31% lower (*P* < 0.05) than those in the R_H group, respectively (Supplementary Table 6). In contrast, the relative abundance of Ascomycota in the root tissues of the diseased plants (R_D) was 3.73% higher (*P* < 0.01) than that of the healthy plants (R_H) (Supplementary Table 7).

At the genus level, *Chryseobacterium, Allorhizobium-Neorhizobium-Parahizobium-Rhizobium, Stenotrophomonas, Pseudomonas*, and *Rhodanobacter*were the dominant bacterial communities in the tobacco root tissues, and *Fusarium, Plectosphaerella, Penicillium, Hematonectria*, and *Setophoma* were the dominant fungal communities (Supplementary Figure 4). The results of the multispecies difference tests of the bacteria and fungi in the root tissues of the diseased and healthy plants revealed that the relative abundances of *Chryseobacterium, Allorhizobium-Neorhizobium-Parahizobium-Rhizobium, Stenotrophomonas, Pseudomonas*, and *Pectobacterium* were significantly higher in the root tissues of the diseased plants than in those of the healthy plants (*P* < 0.01), while the relative abundances of bacterial communities such as *Rhodanobacter, Ensifer, Chujaibacter, Lysobacter, Humibacter, Massilia, Burkholderia-Caballeronia-Paraburkholderia, Streptomyces*, and *Sphingopyxis* were lower (*P* < 0.05) compared to those of the healthy plants (Figure 4C). Among the fungal communities, the relative abundances of *Fusarium, Hematonectria*, and *Pyrenochaeta* in the root tissues of the diseased plants were 254.21, 100.02, and 51.87% higher (*P* < 0.05) than those of the healthy plants, respectively, while the relative abundances of *Plectosphaerella, Penicillium*, and *Setophoma* in the root tissues of the diseased plants were 30.53, 94.31, and 37.54% lower (*P* < 0.05) than those of the healthy plants, respectively (Figure 4D). Our results suggested that the compositions of the endophytic bacterial and fungal communities in the root tissues of the diseased and healthy tobacco plants also differed significantly, particularly in the relative abundances of the dominant communities. Overall, regardless of whether in the rhizosphere soil or root tissues, the healthy tobacco plants effectively recruited a larger number of beneficial microbes, while some pathogenic microbial communities, such as *Fusarium, Alternaria*, and *Pectobacterium*, tended to be enriched in the rhizosphere soil and root tissues of the diseased tobacco plants.

### 3.5 Co-occurrence networks of the rhizosphere microbiomes in the diseased and healthy tobacco plants

**Figure 5:**
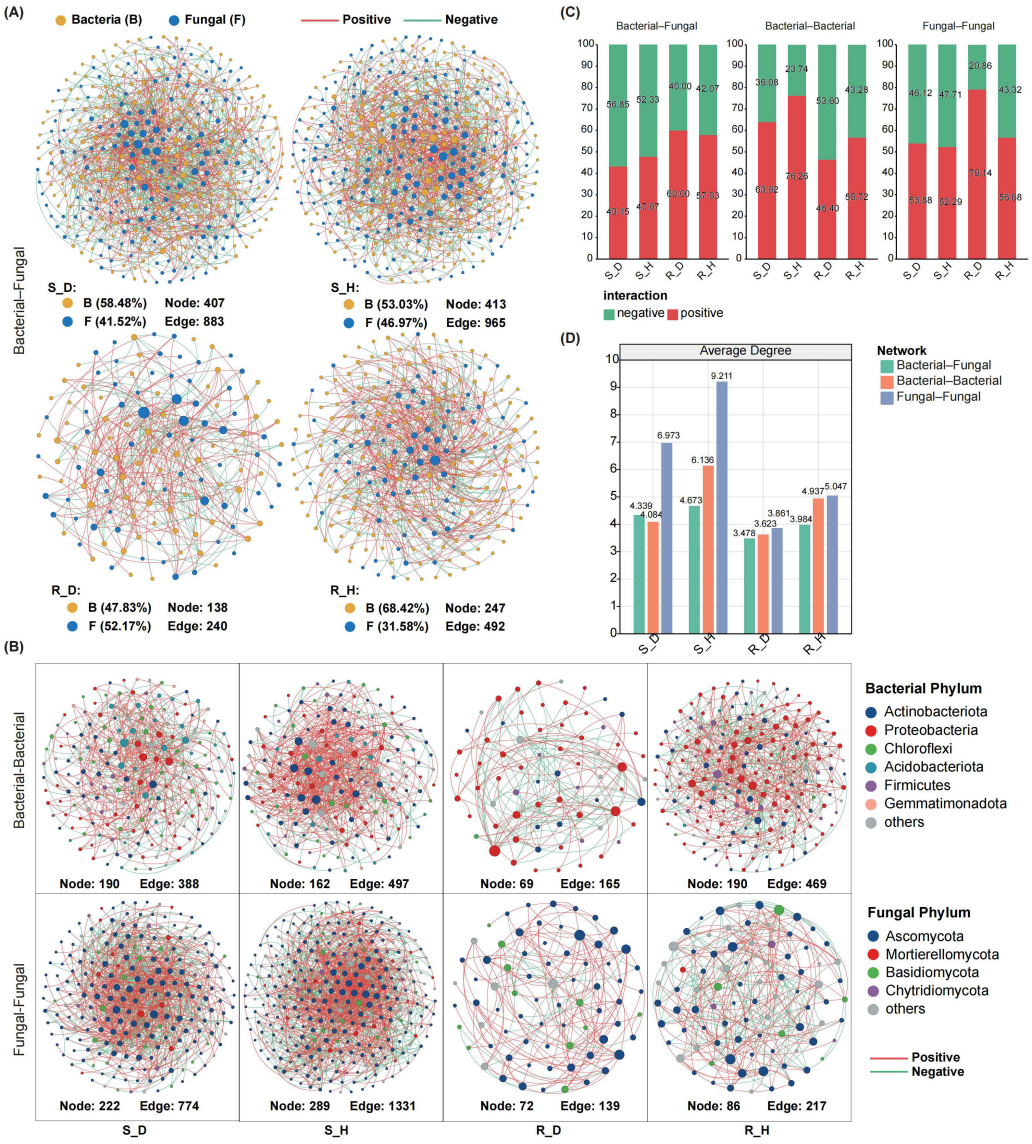
Co-occurrence networks of microbial communities in the rhizosphere soil and root endosphere of tobacco plants under diseased and healthy conditions. **(A)** The inter-domain networks between bacteria and fungi communities in the rhizosphere soil (up) and root endosphere (down) of tobacco plants under diseased and healthy conditions. Both the diseased networks in the rhizosphere soil and root endosphere showed a lower number of nodes and edges than the healthy networks. Edge color represents positive (red) and negative (green) correlations. **(B)** The intra-domain networks of bacterial-bacterial and fungal-fungal in the rhizosphere soil and root endosphere of tobacco plants under diseased and healthy conditions. **(C)** The proportion of bacterial–fungal, bacterial–bacterial, and fungal–fungal correlations in the healthy and diseased networks. Red and green colors of the column indicate positive and negative correlations, respectively. **(D)** Average degree of the healthy and diseased networks. Green, orange and blue represent the bacterial–fungal, bacterial–bacterial, and fungal–fungal networks, respectively.

To study the effects of *Fusarium* root rot on the co-occurrence patterns of tobacco rhizosphere microbial communities, we analyzed the bacteria–fungi interkingdom networks as well as bacteria–bacteria and fungi–fungi intrakingdom networks in the rhizosphere soil and root tissues of diseased and healthy tobacco plants. First, the analysis of bacteria–fungi interkingdom networks revealed that *Fusarium* root rot disrupted their stability. The number of nodes, number of edges, and average connectivity of bacteria–fungi interkingdom networks in both the rhizosphere soil and root tissues of the diseased tobacco plants were lower than those of the healthy plants (Figures 5A, D). In the bacteria–fungi interkingdom network in the rhizosphere soil of the healthy and diseased plants, the proportion of bacterial taxa was higher than that of fungal taxa, the bacteria–fungi interkingdom correlations were mainly negative, and the proportion of negative edges in the diseased network (S_D, 56.85%) was higher than that in the healthy network (S_H, 52.33%). In the root tissues, the proportion of bacterial taxa was higher than that of fungal taxa in the healthy plants; however, in the diseased plants, the proportion of fungal taxa was significantly higher than that in the healthy plants and exceeded that of the bacterial taxa. In the root tissues, the bacteria–fungi interkingdom correlations were mainly positive, and the proportion of positive edges in the diseased network (R_D, 60.00%) was higher than that in the healthy network (R_H, 57.93%) (Figure 5C).

Intrakingdom network analysis showed that, compared with healthy plants, *Fusarium* root rot significantly reduced the complexity of bacteria–bacteria and fungi–fungi intrakingdom networks in both the rhizosphere soil and root tissues of tobacco plants (based on the number of nodes, number of edges, and the average degree) (Figures 5B, D). In the rhizosphere soil, the bacteria–bacteria and fungi–fungi intrakingdom correlations in both the healthy and diseased networks were mainly positive; the diseased bacterial network (S_D) had a lower proportion of positive edges and a higher proportion of negative edges than the healthy network (S_H), while the fungal network showed the opposite pattern. In the root tissues, there were mainly negative intrakingdom correlations of the bacterial network in the diseased plants, with a higher proportion of negative edges than that in the healthy plants. In contrast, there were mainly positive intrakingdom correlations of the bacterial network in the healthy plants. In both healthy and diseased plants, there were mainly positive intrakingdom correlations of the fungal network in the root tissues, with the proportion of positive edges in the diseased network (R_D, 79.14%) being higher than that in the healthy network (R_H, 56.68%) (Figure 5C).

### 3.6 Functional composition of the rhizosphere microbiomes in the diseased and healthy tobacco plants

To further clarify the ecological functions of the bacterial and fungal communities in the rhizosphere of diseased and healthy tobacco plants, we performed metagenomic sequencing on rhizosphere soil samples from three diseased plants and three healthy plants. After quality control, a total of 523,193,998 clean reads were obtained from the six rhizosphere soil samples, and these reads were assembled into an average of approximately 821,603 contigs (Supplementary Table 8). A total of 30,216 bacterial and 806 fungal species were annotated on the basis of the metagenomic data. Consistent with the amplicon sequencing data, the bacterial communities were mainly composed of *Actinomycetota, Pseudomonadota, Acidobacteriota, Gemmatimonadota*, and *Chloroflexota* species, and the fungal communities were primarily composed of *Ascomycota, Mucoromycota*, and *Oomycota* species.

**Figure 6:**
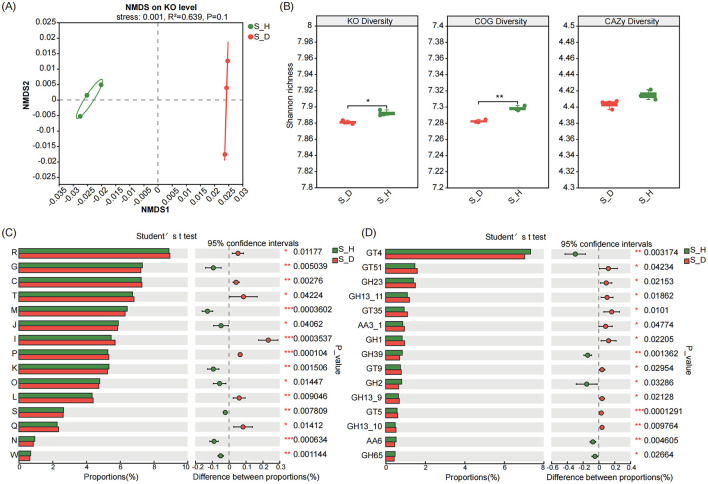
Functional profiles of microbiomes in the rhizosphere soil of tobacco plants under diseased and healthy conditions. **(A)** NMDS ordinations of functional genes based on Bray-Curtis distance matrices of KO functional genes show the distinct functions of microbial communities in the rhizosphere soil of tobacco plants under diseased and healthy conditions. **(B)** The boxplot shows the functional diversity (including KO, COG and CAZy) of the rhizosphere microbiomes of tobacco plants under diseased and healthy conditions. **(C, D)** Differential abundance analysis of COG **(C)** and CAZy **(D)** functional genes of the rhizosphere microbiomes of of tobacco plants under diseased and healthy conditions. * Stands for 0.01 ≤ *p* < 0.05, ** stands for 0.001 ≤ *p* < 0.01, and *** stands for *p* < 0.001 according to Student's *t*-test.

Non-metric multidimensional scaling (NMDS) ordination analysis based on different functional levels revealed a clear separation between diseased and healthy soil samples along the NMDS1 axis and located on the left and right sides of the NMDS1 axis, respectively, indicating that the Kyoto Encyclopedia of Genes and Genomes (KEGG (based on the KEGG Orthology (KO) level), Clusters of Orthologous Genes (COG), and Carbohydrate-Active Enzymes (CAZy)-related functions of the microbial communities in the rhizosphere soil of the diseased tobacco plants shifted significantly compared to those in the healthy plants (*R*^2^ = 0.639 for KO, *R*^2^ = 0.685 for COG, and *R*^2^ = 0.586 for CAZy; *P* = 0.1) (Figure 6A; Supplementary Figure 5). We further found that the functional diversity of KO, COG, and CAZy in the rhizosphere soil of the diseased tobacco plants was lower than that in the healthy plants (based on the Shannon index); in particular, the KO and COG functional diversity was significantly lower than that in the healthy plants (*P* < 0.05) (Figure 6B).

We performed differential abundance analysis to determine the functional characteristics of the rhizosphere microbiomes of the diseased and healthy tobacco plants. First, in terms of COG functions, the relative abundances of genes in the carbohydrate transport and metabolism (COG_G), cell wall/membrane/envelope biogenesis (COG_M), translation, ribosome structure and biogenesis (COG_J), transcription (COG_K), posttranslational modification, protein turnover, chaperone (COG_O), and cell movement (COG_N) modules were significantly lower in the diseased plants than in the healthy plants (*P* < 0.05). In contrast, the relative abundances of genes in the energy production and conversion (COG_C), signal transduction mechanisms (COG_T), lipid transport and metabolism (COG_I), inorganic ion transport and metabolism (COG_P), replication, recombination and repair (COG_L), secondary metabolites biosynthesis, transport and catabolism (COG_Q) modules were significantly higher in the diseased plants than in the healthy plants (*P* < 0.05) (Figure 6C). In terms of CAZy functions, the relative abundances of genes such as sucrose synthase (GT4), beta-xylosidase (GH39), beta-galactosidase (GH2), 1,4-benzoquinone reductase (AA6), and alpha, alpha-trehalase (GH65) were significantly lower in the diseased plants than in the healthy plants (*P* < 0.05), while the relative abundances of genes such as murein polymerase (GT51), lysozyme type G (GH23), glycogen or starch phosphorylase (GT35), beta-glucosidase (GH1), lipopolysaccharide N-acetyl glucosyltransferase (GT9), and UDP-Glc: glycogen glucosyltransferase (GT5) were significantly higher than those in the healthy plants (P < 0.05) (Figure 6D).

**Figure 7:**
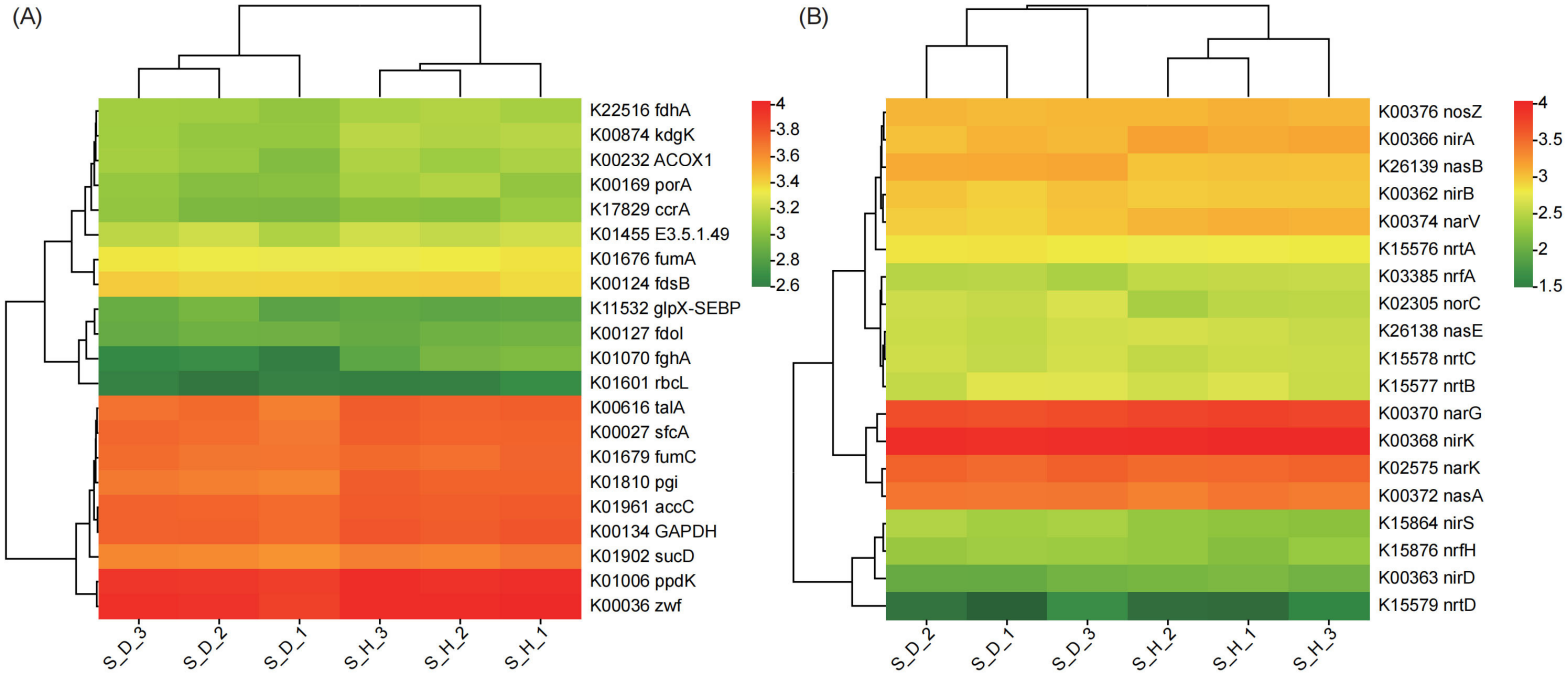
Heatmap exhibiting the relative abundance of functional genes (based on KO) involved in carbon **(A)** and nitrogen **(B)** metabolism in the rhizosphere microbial communities of tobacco plants under diseased and healthy conditions. Each row of the heatmap corresponds to a specific gene, while each column represents a different sample. The colors in the heatmap, red representing higher relative abundance levels of the gene in that sample, and green representing lower relative abundance levels, indicate the variation in gene relative abundance.

Considering that soil microbial communities participate in the material cycle and transformation of the soil mainly through their metabolic activities and are closely related to the soil nutrient cycle, we further analyzed the changes in the relative abundance (based on the KO level) of genes involved in C and N metabolism. In the heatmap of genes related to C and N metabolism, the samples of the S_D group and S_H group were located on different branches, suggesting that the gene expression patterns related to C and N metabolism in the rhizosphere microbial communities differed significantly between the diseased and healthy plants. We found that the relative abundances of many genes related to C metabolism in the diseased plants were lower those in the healthy plants, especially for genes such as S-formylglutathione hydrolase (fghA), glucose-6-phosphate isomerase (pgi), 2-dehydro-3-deoxygluconokinase (kdgK), acyl-CoA oxidase (ACOX1), pyruvate ferredoxin oxidoreductase alpha subunit (porA), glucose-6-phosphate 1-dehydrogenase (zwf), and ribulose-bisphosphate carboxylase large chain (rbcL) (Figure 7A).

Among the genes related to N metabolism, the relative abundances of genes in the nitrate/nitrite transport system, including ATP-binding protein (nrtC), permease protein (nrtB), and substrate-binding protein (nrtA), were higher in the diseased plants than in the healthy plants; in addition, the relative abundances of genes such as nitrite reductase [NAD(P)H] large subunit (nasB), nitric oxide reductase subunit C (norC), assimilatory nitrate reductase catalytic subunit (nasA), cytochrome c nitrite reductase small subunit (nrfH), and nitrite reductase (NO-forming)/hydroxylamine reductase (nirS) were also higher than those in the healthy plants. In contrast, the relative abundances of genes such as ferredoxin-nitrite reductase (nirA), nitrate reductase gamma subunit (narV), nitrite reductase (cytochrome c-552) (nrfA), nitrate reductase/nitrite oxidoreductase, alpha subunit (narG), and nitrite reductase (NO-forming) (nirK) were lower in the diseased plants than in the healthy plants (Figure 7B). In conclusion, the above results indicate that, compared with the healthy state, the relevant functions of the tobacco rhizosphere soil microbial community in the state of *Fusarium* root rot disease have undergone a relatively large shift, and the functional diversity of the rhizosphere microorganisms is significantly reduced. In the diseased state, genes in the tobacco rhizosphere soil microbial community related to energy production and conversion, signal transduction mechanisms, replication, recombination and repair, transport and catabolism, as well as genes related to N metabolism are more abundant than those in the healthy state, but the functional genes related to C metabolism are significantly reduced.

## 4 Discussion

*Fusarium* root rot in tobacco is a soil-borne vascular disease that severely harms tobacco production. Pathogenic fungi can survive in soil for a long period of time, greatly increasing the difficulty of disease control. The rhizosphere is a key microzone for plant–soil–microbe interactions (Zheng et al., 2022), and the rhizosphere microbiota is considered the first line of defense against soil–borne pathogens, serving as a crucial determinant of plant health (Li et al., 2021; Wen et al., 2022; Yang et al., 2023). In this study, we systematically analyzed the differences in the composition and function of rhizosphere soil and endophytic microbial communities in tobacco plants with *Fusarium* root rot and healthy plants, and we further elucidated the interactions between microbial communities, aiming to elucidate the changes in the microbial communities in the host plant rhizosphere and root endosphere under the stress of soil-borne fungal pathogens, providing a theoretical basis for the future application of related microbial communities in the sustainable improvement of agricultural productivity.

Consistent with previous studies, we found in the present study that microbial diversity gradually decreased from the rhizosphere to the root endosphere (Edwards et al., 2015), but both bacterial and fungal community structures and diversities in the rhizosphere soil and root endosphere showed dynamic changes in response to *Fusarium* infection. Based on PCoA using the Bray–Curtis distance algorithm, we found that the bacterial and fungal communities in the tobacco rhizosphere soil and root endosphere of tobacco plants were separated along the first principal component in both the diseased and healthy plants, suggesting that the microbial community structures in the rhizosphere soil and root endosphere of tobacco plants differed significantly between the two states. We further found that *Fusarium* infection increased the diversity and abundance of bacterial communities in tobacco rhizosphere soil but reduced those of fungal communities (based on the Shannon and Chao 1 indices); within root tissues, the diversities of both bacterial and fungal communities (Shannon index) were significantly reduced. The simultaneous decrease in the diversity of endophytic bacterial and fungal communities in root tissues may result from pathogenic fungal invasion and colonization in root tissues. The production of toxins by *Fusarium* pathogens during growth, development, and metabolism (Lievens et al., 2008), as well as their competition with native bacterial and fungal communities in root tissues for niches and nutrients, may contribute to the decreased diversity of native bacterial and fungal communities (Tan et al., 2022). The completely opposite changes in the diversity and abundance of bacterial and fungal communities in the rhizosphere soil were also the result of the dynamic response of native species in the tobacco rhizosphere to pathogen infection; under pathogen stress, plants usually secrete specific root exudates (e.g., sugars, amino acids, organic acids, fatty acids, and secondary metabolites) to attract soil microbes to the plant rhizosphere. On the one hand, this can increase the diversity of native species and enhance the collective competitiveness of the community (Trivedi et al., 2020; Yin et al., 2021); on the other hand, these exudates can attract beneficial microbes to protect the host plant by inhibiting soil-borne pathogens or inducing systemic resistance in plants (Bai et al., 2022). Admittedly, this attraction is not always positive, as more soil-borne pathogens or harmful microbes may also accumulate in the rhizosphere, resulting in an imbalance in the rhizosphere microecosystem and aggravating the incidence of disease (Nadarajah and Abdul Rahman, 2021). Therefore, we further analyzed the species composition of the bacterial and fungal communities in the rhizosphere soil and the root endosphere of diseased and healthy tobacco plants.

Similar to most research findings, regardless of the disease state, the microbial communities in the rhizosphere soil and the root endosphere of tobacco plants were mainly composed of bacteria such as Actinobacteria, Proteobacteria, Acidobacteria, and Firmicutes and fungi such as Ascomycota and Basidiomycota, but some dominant bacterial and fungal communities exhibited significant differences between the diseased and healthy plants. We found that in the rhizosphere soil, the relative abundances of Proteobacteria, Acidobacteria, Firmicutes, Ascomycota, and Basidiomycota were higher in the diseased plants than in the healthy plants. Among them, Proteobacteria plays an important role in the soil C, S, and N cycles (Wang et al., 2017); Firmicutes, Ascomycota, and Basidiomycota are important decomposers in the C cycle, as they can secrete hydrolytic enzymes to break down organic matter (e.g., cellulose, lignocellulose, and lignin in plant litter) into smaller molecules, playing a key role in plant litter degradation and promoting the soil C cycle (Wang et al., 2017; Wei et al., 2018; Wang et al., 2020). After tobacco plants become diseased, the functional microbial communities related to soil nutrient cycling in rhizosphere soil increased in abundance, which may be related to the damage to vascular bundles and root tissues in the diseased plants, reducing their ability to uptake nutrients from the soil. In this study, we also found that the contents of organic matter, available nitrogen, available phosphorus, and available potassium in the rhizosphere soil of diseased plants were higher than those in the rhizosphere soil of healthy plants. In addition, most Actinomycetota have been reported to produce antibiotics, inhibit plant pathogens, and control plant diseases (Barka et al., 2015), but here, we noted a significant decrease in the relative abundance of Actinomycetota in the diseased plants compared to that in the healthy plants. At the genus level, we observed higher relative abundances of bacteria (*Arthrobacter* and *Sphingomonas*) and fungi (*Alternaria, Fusarium, Filobasidium*, and *Cladosporium*) in the rhizosphere soil of diseased tobacco plants. Among them, the significantly more abundant *Alternaria* and *Fusarium* have been reported to cause diseases such as brown spot disease (Chen et al., 2022) and root rot (Yang et al., 2020; Tan et al., 2022) in tobacco plants, and *Filobasidium* species have been reported to contain plant cell wall-degrading CAZymes (Guerreiro et al., 2022). Therefore, although the diversity of fungal communities in the rhizosphere soil of tobacco plants was significantly higher in the diseased plants, the composition of the microbial communities tended to form pathological combinations, potentially further aggravating the disease. In contrast, the relative abundances of beneficial microbial communities such as *Streptomyces, Mortierella*, and *Penicillium* were significantly lower in the diseased plants than in the healthy plants. Among them, *Streptomyces* is well known for its strong ability to produce antibiotics (Schlatter et al., 2009). Species of the genus *Mortierella* have been reported to be related to soil disease inhibition, potentially suppressing diseases caused by *Fusarium* and participating in the transformation of P in the soil (Wang et al., 2022). *Penicillium* can participate in the decomposition of organic matter, promote the cycling of various elements such as C, N, and P, and degrade a variety of environmentally harmful substances. These results further indicate that these known beneficial microorganisms may be rejected by dominant pathogens or defeated by other dominant and harmful microbes with increased mobility, thereby causing or exacerbating the successful invasion of root rot pathogens (Tan et al., 2022).

Similarly, in the diseased plants, we also observed a shift from microbial community combination to pathological combination in the root tissues. At the phylum level, the relative abundances of *Actinobacteria* and *Firmicutes* in the root tissues of diseased plants were significantly lower than those in healthy plants, whereas the relative abundance of Ascomycota was significantly higher in the diseased plants than in the healthy plants. At the genus level, the relative abundances of *Chryseobacterium, Allorhizobium-Neorhizobium-Parahizobium-Rhizobium, Stenotrophomonas, Pseudomonas, Pectobacterium, Fusarium*, and *Hematonectria* were significantly higher in the root tissues of the diseased plants, whereas the relative abundances of *Rhodanobacter, Lysobacter, Streptomyces, Plectosphaerella, Penicillium*, and *Setophoma* were significantly lower than those in the healthy root tissues. Here, we noted that the relative abundance of *Fusarium* in the root tissues of the diseased plants was 254.21% higher than in those of the healthy plants, suggesting that the successful invasion and colonization of root rot pathogens is a major reason for the changes in the structure and composition of root endosphere microbial communities.

To further elucidate the interactions of related microbial communities, we performed microbial co-occurrence network analysis. We found that *Fusarium* root rot disrupted the stability of interkingdom ecological networks. In the diseased plants, the number of nodes, number of edges, and average connectivity of bacteria–fungi interkingdom networks in both the rhizosphere soil and the root endosphere were lower than those in the healthy plants. In the rhizosphere soil, the bacteria–fungi interkingdom correlations were mainly negative, and the proportion of negative edges in the diseased network (S_D, 56.85%) was higher than that in the healthy network (S_H, 52.33%). In the root endosphere, the bacteria–fungi interdomain correlations were mainly positive, and the proportion of positive edges in the diseased network (R_D, 60.00%) was higher than that in the healthy network (R_H, 57.93%). The cooperative and competitive interactions between microbial species can affect community stability. Negative interactions represent ecological competition between bacteria and fungi, whereas positive interactions indicate weaker competition between bacteria and fungi, potentially shifting toward cooperative or mutualistic relationships (Faust and Raes, 2012; Coyte et al., 2015). In the rhizosphere soil, competition dominated the relationships between bacteria and fungi, possibly because the two usually compete for plant-derived substrates (Boer et al., 2005). In the root tissues, cooperation dominated the relationships between bacteria and fungi, which can be explained by the invasion of pathogenic fungi, which require establishing mutualistic relationships with other species to promote their successful colonization (Van Elsas et al., 2002). In addition, we found that in the diseased plants, the complexity of fungi–fungi intrakingdom networks in both the rhizosphere soil and the root endosphere was higher than that of bacteria–bacteria intrakingdom networks; the number of nodes, number of edges, and average connectivity of the fungi–fungi intrakingdom networks were higher than those of the bacterial networks; and the correlations within the fungal kingdom were mainly positive, with the proportion of positive correlations in the diseased root tissues reaching as high as 79.14%. These findings underscore the ecological importance of the fungal taxa and suggest that in the diseased plants, the fungal taxa in the tobacco rhizosphere and root endosphere mainly engage in cooperative interactions, possibly because pathogenic fungi need to establish mutualistic relationships with other fungal species after invasion (Tan et al., 2022). Our results further support that the changes in the composition and relative abundances of dominant bacterial and fungal communities in the tobacco rhizosphere soil and the root endosphere in the diseased plants were all the result of interactions between the root rot pathogens and native microorganisms.

In addition to community composition, this study revealed that the ecological functions of the microbial communities in the tobacco plant rhizosphere soils also greatly differed between the healthy and diseased plants, with KO, COG, and CAZy functional diversity in the tobacco rhizosphere soil being lower in the diseased plants than in the healthy plants. The rhizosphere of the diseased tobacco plants showed higher abundances of microbial functional genes involved in energy production and conversion, signal transduction mechanisms, lipid transport and metabolism, replication, recombination and repair, and biosynthesis, transport, and catabolism of secondary metabolites, while there were fewer microbial functional genes related to carbohydrate transport and metabolism, cell wall/membrane/envelope biogenesis, and cell motility. It is well known that soil microbes play an important role in soil nutrient cycling, and soil physicochemical properties, such as nutrient availability, also affect the composition of plant root microbial communities (Bai et al., 2022). Changes in the metabolism of C, N, and P in the soil may lead to competition for nutrients among different microbial groups, affecting the balance between beneficial microbes and soil-borne pathogens, thereby resulting in disease outbreaks (Wang et al., 2020; Cai et al., 2021). Here, we found that the relative abundances of genes associated with C metabolism in the diseased plants were lower than those in the healthy plants, especially the significantly reduced relative abundance of rbcL, which is associated with C fixation; however, the relative abundances of genes associated with N degradation, such as nasB and nasA, were higher in the diseased plants than in the healthy plants. Overall, the rhizosphere microbial communities of the diseased tobacco plants played a significant role in basic biological metabolism, energy production and conversion, signal transduction, and N metabolism, but the functions involved in C metabolism were weakened, suggesting an unfavorable shift in the tobacco rhizosphere microecology. However, there are certain limitations in exploring the functions and interactions of microbial communities under Fusarium infection solely based on metagenomic analysis. In future work, we will combine isolation, cultivation, and synthetic community construction techniques to further validate the functional roles of beneficial microbial communities in tobacco's resistance to *Fusarium* infection, while laying the foundation for the development and utilization of beneficial microbial consortia as biocontrol agents or antimicrobial metabolites.

## 5 Conclusion

In this study, we conclude that *Fusarium* infection leads to simultaneous changes in bacterial and fungal communities in the rhizosphere soil and root tissues of tobacco plants, disrupting the stability of the bacterial-fungal interdomain ecological network. The rhizosphere microbial community of diseased tobacco plants shifted toward a pathological combination, with significant increases in the relative abundance of harmful microorganisms such as *Alternaria, Fusarium*, and *Filobasidium*, while the relative abundance of beneficial microorganisms such as *Lysobacter, Streptomyces, Mortierella*, and *Penicillium* significantly decreases. These findings further deepen our understanding of the changes in the microbial communities in the host plant rhizosphere and root endosphere under the stress of soil-borne fungal pathogens and lay a theoretical foundation for the comprehensive utilization of related microbial communities and the development of prevention and control strategies for soil-borne fungal diseases.

## Data Availability

The datasets presented in this study can be found in online repositories. The names of the repository/repositories and accession number(s) can be found below: https://www.ncbi.nlm.nih.gov/, PRJNA1173500 and https://www.ncbi.nlm.nih.gov/, PRJNA1173517.
