# Supplementary material for: Changes in the community composition and function of the rhizosphere microbiome in tobacco plants with Fusarium root rot
Source: Front Microbiol. 2025 Apr 9;16:1512694. doi: 10.3389/fmicb.2025.1512694 (PMC12023262; doi:10.3389/fmicb.2025.1512694)
Supplement: Supplementary file 1 [file Data_Sheet_1.docx]

**<Supplementary materials>**


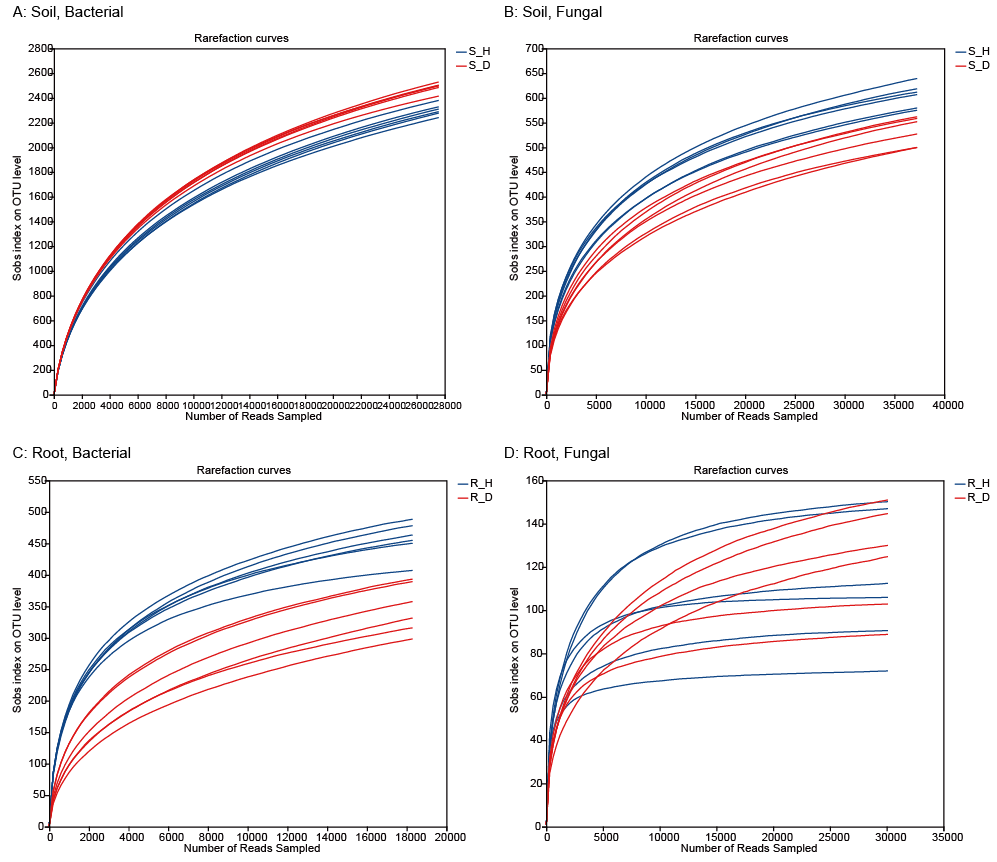


**Figure S1** Rarefaction curves generated from amplification of bacterial (**A**, **C**) and fungal (**B**, **D**) 16S and ITS genes of rRNA, respectively.


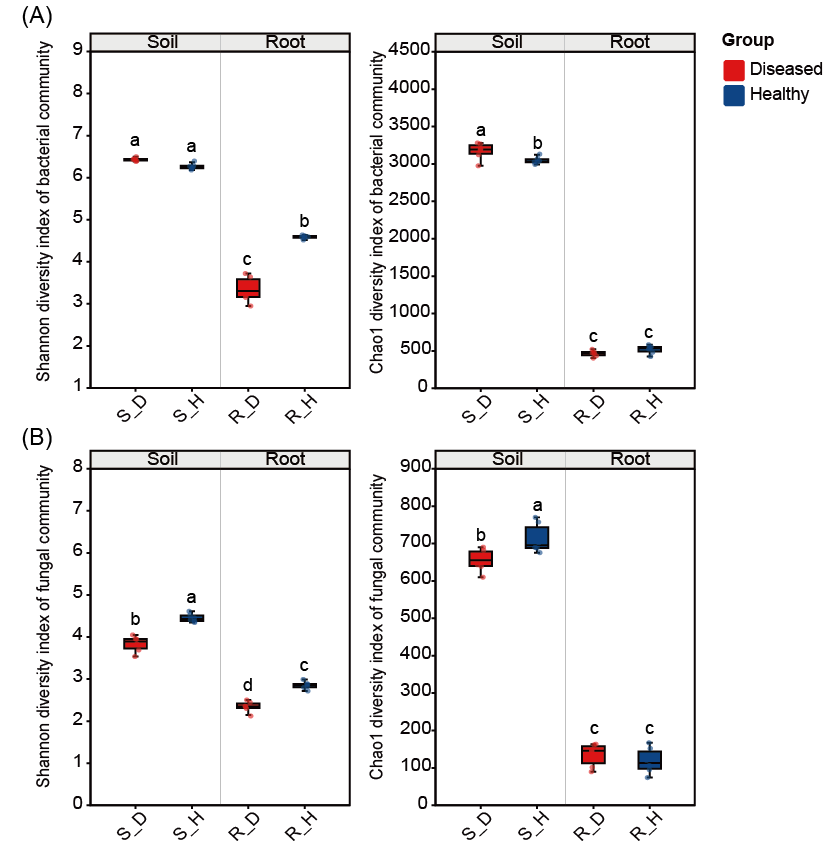


**Figure S2** Shannon and Chao1 diversity indices of the bacterial (**A**) and fungal (**B**) communities in the rhizosphere soil and root endosphere of tobacco plants under diseased and healthy conditions.


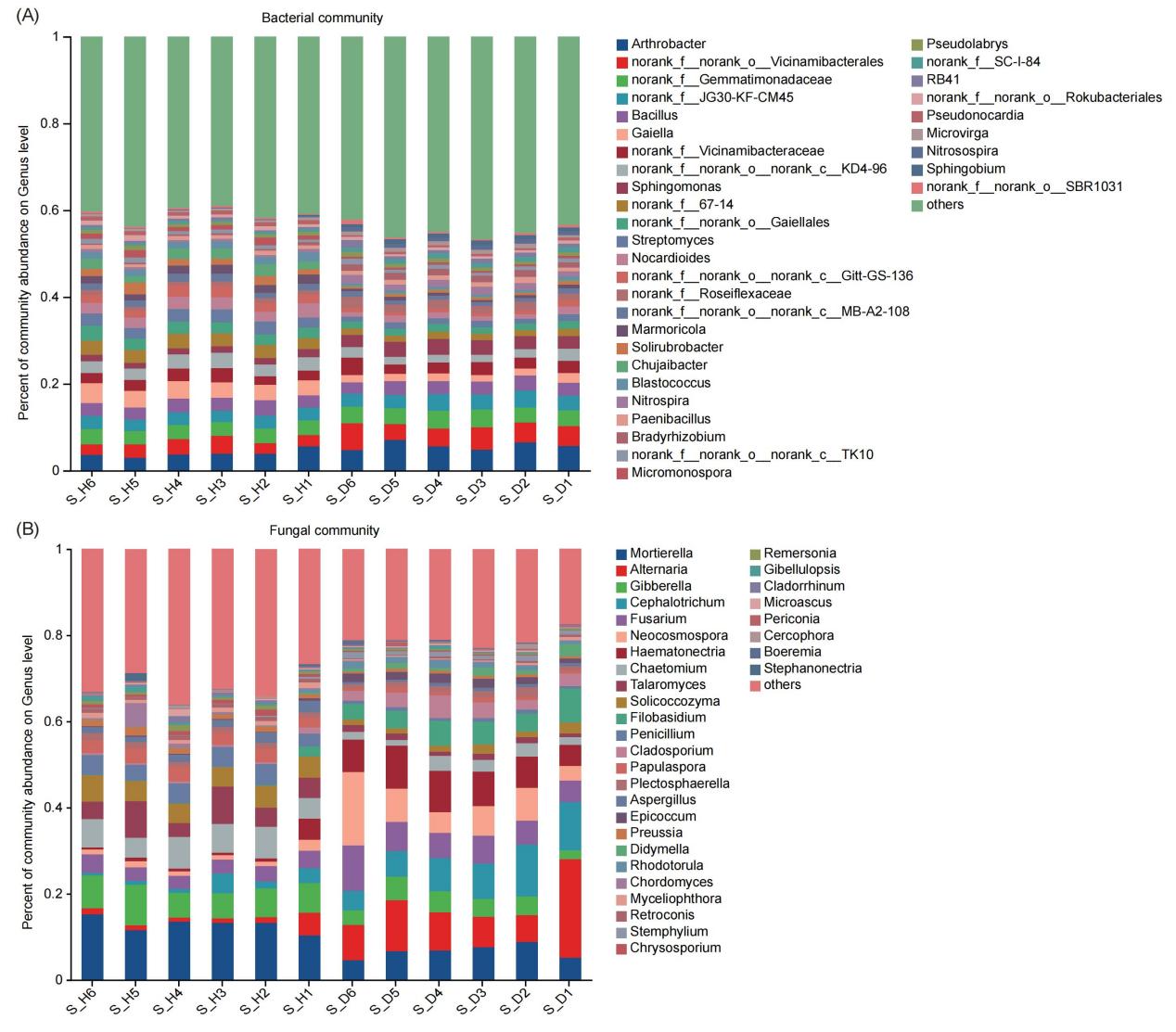


**Figure S3** The bar plots of relative abundance illustrate the composition of bacterial (**A**) and fungal (**B**) communities in the rhizosphere soil at the genus level under diseased and healthy conditions. Low abundance genera with less than 1% of the total sequences across all samples are grouped into “Other”.


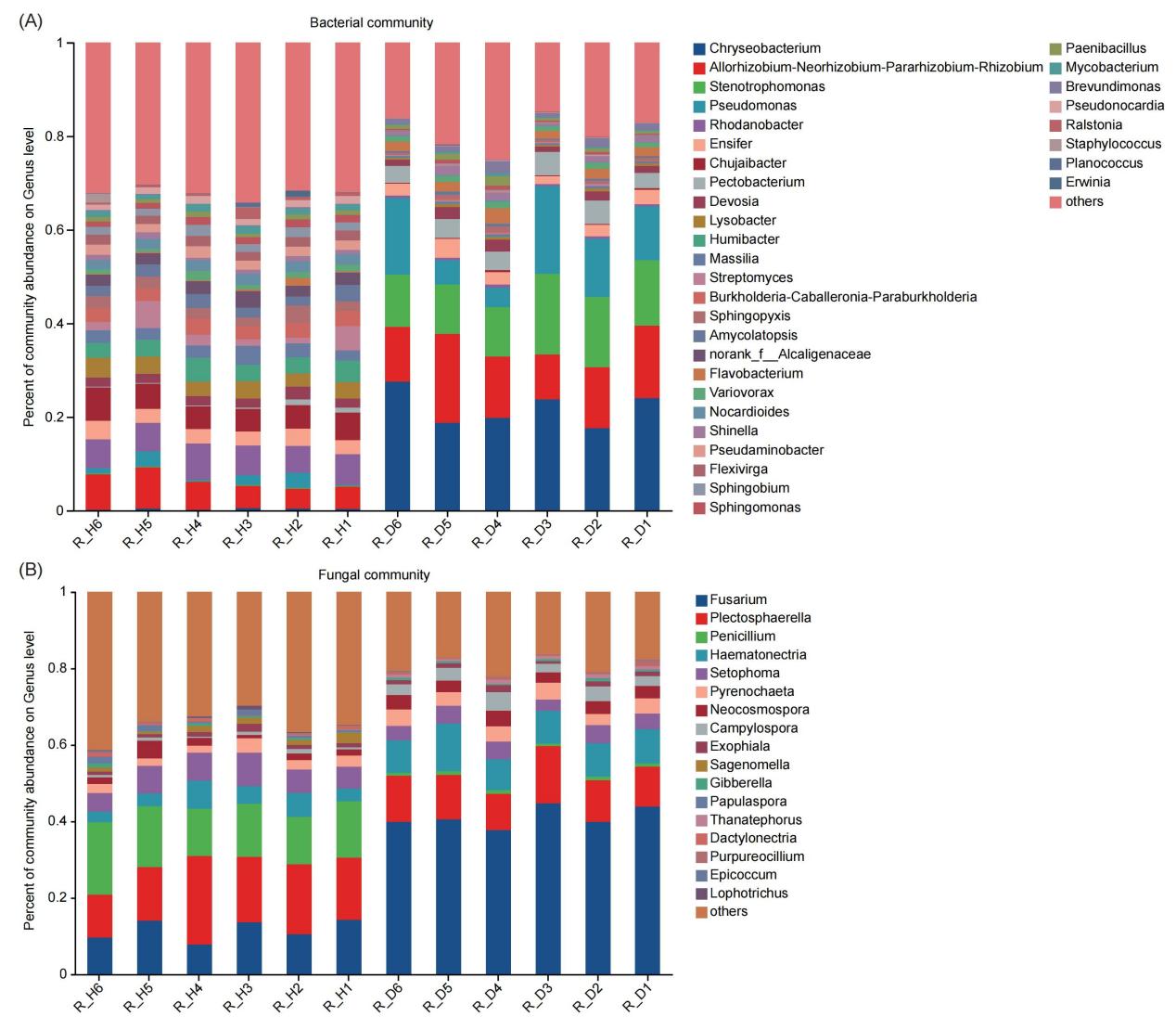


**Figure S4** The bar plots of relative abundance illustrate the composition of bacterial (**A**) and fungal (**B**) communities in the root endosphere at the genus level under diseased and healthy conditions. Low abundance genera with less than 1% of the total sequences across all samples are grouped into “Other”.


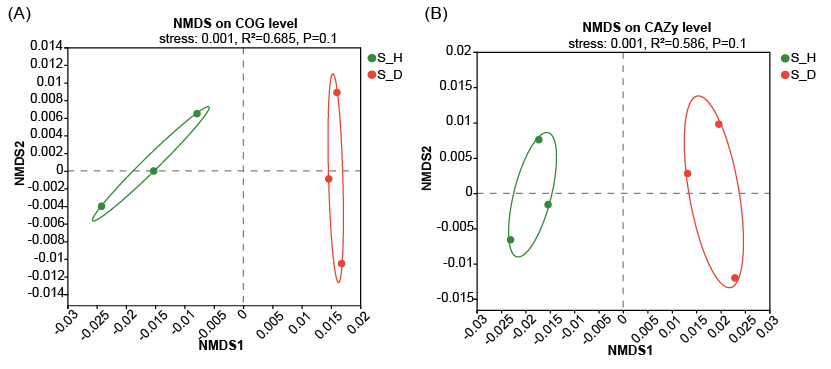


**Figure S5** NMDS ordinations of functional genes based on Bray-Curtis distance matrices of COG (**A**) and CAZy (**B**) functional genes show the distinct microbial communities' function in the rhizosphere soil of tobacco plants under diseased and healthy conditions.

| Table S1 The physiochemical properties of the soil used in the experiment | | | | | |
| --- | --- | --- | --- | --- | --- |
| Samples | pH | OM (g/kg) | AN (mg/kg) | AP (mg/kg) | AK (mg/kg) |
| S_H | 6.25±0.03 | 20.98±0.67 | 159.58±0.97 | 37.27±0.49 | 203.06±0.48 |
| S_D | 5.83±0.01 | 26.28±0.30 | 172.51±0.84 | 42.21±0.33 | 254.68±0.76 |

| Table S2 Statistical analysis of amplicon sequencing results for bacterial and fungal communities in the rhizosphere soil of tobacco plants | | | | | | |
| --- | --- | --- | --- | --- | --- | --- |
|  | Sample | Seq_num | Base_num | Mean_length | Min_length | Max_length |
| Bacterial | S_D1 | 33439 | 13904657 | 415.821556 | 338 | 452 |
|  | S_D2 | 35373 | 14718091 | 416.082634 | 282 | 476 |
|  | S_D3 | 40406 | 16832491 | 416.583948 | 265 | 508 |
|  | S_D4 | 48676 | 20251449 | 416.045875 | 234 | 476 |
|  | S_D5 | 48735 | 20260440 | 415.726685 | 246 | 477 |
|  | S_D6 | 41127 | 17127127 | 416.444842 | 302 | 497 |
|  | S_H1 | 47664 | 19827435 | 415.983447 | 262 | 521 |
|  | S_H2 | 47746 | 19889176 | 416.562141 | 210 | 509 |
|  | S_H3 | 45459 | 18948314 | 416.822059 | 250 | 487 |
|  | S_H4 | 47928 | 19986308 | 417.006927 | 231 | 505 |
|  | S_H5 | 46204 | 19270421 | 417.072569 | 203 | 457 |
|  | S_H6 | 49789 | 20746432 | 416.687059 | 209 | 490 |
| fungal | S_D1 | 56261 | 13296424 | 236.334655 | 143 | 516 |
|  | S_D2 | 49118 | 11549911 | 235.146199 | 142 | 514 |
|  | S_D3 | 42092 | 9860474 | 234.260049 | 142 | 512 |
|  | S_D4 | 40858 | 9553903 | 233.831881 | 140 | 502 |
|  | S_D5 | 38787 | 9094447 | 234.471524 | 142 | 527 |
|  | S_D6 | 43707 | 10107379 | 231.253094 | 140 | 511 |
|  | S_H1 | 50075 | 11890560 | 237.455017 | 142 | 516 |
|  | S_H2 | 54422 | 13091088 | 240.54772 | 143 | 514 |
|  | S_H3 | 49970 | 12041873 | 240.982049 | 143 | 516 |
|  | S_H4 | 45866 | 11121148 | 242.470414 | 140 | 517 |
|  | S_H5 | 44593 | 10628281 | 238.339672 | 143 | 511 |
|  | S_H6 | 43676 | 10417012 | 238.506548 | 142 | 525 |

| Table S3 Statistical analysis of amplicon sequencing results for endophytic bacteria and fungal communities in roots of tobacco plants | | | | | | |
| --- | --- | --- | --- | --- | --- | --- |
|  | Sample | Seq_num | Base_num | Mean_length | Min_length | Max_length |
| Bacterial | R_D1 | 40069 | 15066083 | 376.003469 | 204 | 498 |
|  | R_D2 | 34948 | 13142658 | 376.063237 | 214 | 411 |
|  | R_D3 | 42970 | 16153551 | 375.926251 | 204 | 504 |
|  | R_D4 | 38963 | 14637586 | 375.679131 | 203 | 463 |
|  | R_D5 | 41754 | 15711027 | 376.275974 | 204 | 492 |
|  | R_D6 | 38267 | 14376383 | 375.686179 | 214 | 390 |
|  | R_H1 | 41695 | 15750392 | 377.752536 | 218 | 443 |
|  | R_H2 | 38071 | 14365230 | 377.327362 | 214 | 406 |
|  | R_H3 | 37540 | 14156832 | 377.113266 | 217 | 417 |
|  | R_H4 | 40932 | 15455668 | 377.593765 | 206 | 404 |
|  | R_H5 | 40396 | 15253745 | 377.605332 | 206 | 422 |
|  | R_H6 | 38957 | 14712412 | 377.657725 | 214 | 490 |
| fungal | R_D1 | 42961 | 9883867 | 230.066037 | 143 | 514 |
|  | R_D2 | 38253 | 8844100 | 231.200167 | 158 | 512 |
|  | R_D3 | 45730 | 10464462 | 228.831445 | 185 | 510 |
|  | R_D4 | 35550 | 8266183 | 232.522729 | 172 | 529 |
|  | R_D5 | 46533 | 10683499 | 229.589732 | 155 | 517 |
|  | R_D6 | 44654 | 10294724 | 230.544274 | 141 | 511 |
|  | R_H1 | 37403 | 8965257 | 239.693527 | 147 | 512 |
|  | R_H2 | 32308 | 7684834 | 237.861644 | 144 | 513 |
|  | R_H3 | 30300 | 7247115 | 239.178713 | 146 | 511 |
|  | R_H4 | 32297 | 7653659 | 236.977397 | 143 | 504 |
|  | R_H5 | 30211 | 7232872 | 239.41187 | 144 | 522 |
|  | R_H6 | 30181 | 7300312 | 241.884364 | 142 | 504 |

| Table S4 Relative abundance of bacterial phyla in rhizosphere soil samples of tobacco plants under diseased and healthy conditions | | | | | |
| --- | --- | --- | --- | --- | --- |
| Species name | S_D-Mean(%) | S_D-Sd(%) | S_H-Mean(%) | S_H-Sd(%) | P_value |
| Actinobacteriota | 25.69 | 2.690 | 41.90 | 1.246 | 0.005 |
| Proteobacteria | 23.75 | 1.629 | 17.58 | 0.600 | 0.005 |
| Chloroflexi | 13.51 | 1.450 | 13.04 | 0.726 | 0.689 |
| Acidobacteriota | 11.78 | 2.128 | 8.19 | 1.306 | 0.008 |
| Firmicutes | 8.84 | 0.851 | 7.49 | 0.587 | 0.013 |
| Gemmatimonadota | 5.33 | 0.346 | 4.48 | 0.190 | 0.005 |
| Myxococcota | 2.38 | 0.240 | 2.04 | 0.301 | 0.128 |
| Bacteroidota | 2.25 | 0.532 | 1.19 | 0.200 | 0.008 |
| Patescibacteria | 1.70 | 0.165 | 0.53 | 0.072 | 0.005 |
| Nitrospirota | 1.59 | 0.295 | 0.62 | 0.093 | 0.005 |
| Methylomirabilota | 0.67 | 0.112 | 0.99 | 0.351 | 0.093 |
| others | 2.51 | 0.197 | 1.96 | 0.439 | 0.025 |

| Table S5 Relative abundance of fungal phyla in rhizosphere soil samples of tobacco plants under diseased and healthy conditions | | | | | |
| --- | --- | --- | --- | --- | --- |
| Species name | S_D-Mean(%) | S_D-Sd(%) | S_H-Mean(%) | S_H-Sd(%) | P_value |
| Ascomycota | 78.87 | 2.626 | 75.68 | 2.119 | 0.045 |
| Mortierellomycota | 6.65 | 1.426 | 13.49 | 1.974 | 0.005 |
| Basidiomycota | 9.59 | 1.915 | 6.79 | 1.426 | 0.020 |
| unclassified_k__Fungi | 3.58 | 0.885 | 1.60 | 0.279 | 0.005 |
| Chytridiomycota | 1.16 | 0.256 | 2.23 | 0.556 | 0.013 |
| others | 0.15 | 0.066 | 0.20 | 0.053 | 0.093 |

| Table S6 Relative abundance of bacterial phyla in root samples of tobacco plants under diseased and healthy conditions | | | | | |
| --- | --- | --- | --- | --- | --- |
| Species name | R_D-Mean(%) | R_D-Sd(%) | R_H-Mean(%) | R_H-Sd(%) | P_value |
| Proteobacteria | 71.09 | 2.997 | 69.71 | 2.589 | 0.471 |
| Actinobacteriota | 2.81 | 1.096 | 25.40 | 3.398 | 0.005 |
| Bacteroidota | 24.36 | 3.543 | 0.90 | 0.642 | 0.005 |
| Firmicutes | 1.21 | 0.762 | 2.39 | 1.048 | 0.045 |
| others | 0.54 | 0.017 | 1.59 | 0.027 | 0.176 |

| Table S7 Relative abundance of fungal phyla in root samples of tobacco plants under diseased and healthy conditions | | | | | |
| --- | --- | --- | --- | --- | --- |
| Species name | R_D-Mean(%) | R_D-Sd(%) | R_H-Mean(%) | R_H-Sd(%) | P_value |
| Ascomycota | 97.45 | 0.674 | 93.95 | 1.126 | 0.005 |
| Basidiomycota | 1.88 | 0.458 | 2.29 | 0.720 | 0.379 |
| Chytridiomycota | 0.00 | 0.003 | 1.65 | 0.494 | 0.005 |
| others | 0.66 | 0.045 | 2.11 | 0.124 | 0.087 |

| Table S8 Sequencing statistics of the soil metagenomic libraries. | | | | | | | | |
| --- | --- | --- | --- | --- | --- | --- | --- | --- |
| Samples | Raw reads | Raw base  (bp) | Clean reads | Clean base  (bp) | No. Contigs | Contigs bases  (bp) | No. ORFs | Average Length  (bp) |
| S_D_1 | 81322730 | 12279732230 | 79666514 | 12010428830 | 735423 | 394447154 | 879542 | 401.7 |
| S_D_2 | 86314338 | 13033465038 | 84720730 | 12772555905 | 798141 | 431441374 | 959766 | 403.07 |
| S_D_3 | 89806942 | 13560848242 | 88149864 | 13289453029 | 816497 | 440554449 | 981276 | 403.17 |
| S_H_1 | 93783992 | 14161382792 | 91868286 | 13849803699 | 868469 | 491767082 | 1064385 | 413.63 |
| S_H_2 | 90679798 | 13692649498 | 88660014 | 13364149592 | 838377 | 465143284 | 1021139 | 409.59 |
| S_H_3 | 92160184 | 13916187784 | 90128590 | 13584196902 | 872712 | 476766939 | 1055946 | 405.71 |
